# Comprehensive analysis of ferritin subunits expression and positive correlations with tumor-associated macrophages and T regulatory cells infiltration in most solid tumors

**DOI:** 10.18632/aging.202841

**Published:** 2021-04-16

**Authors:** Zhang-Wei Hu, Lin Chen, Ren-Qiang Ma, Fan-Qin Wei, Yi-Hui Wen, Xue-Lan Zeng, Wei Sun, Wei-Ping Wen

**Affiliations:** 1 Department of Otolaryngology, The First Affiliated Hospital, Sun Yat-Sen University, Guangzhou 510080, Guangdong, P.R. China; 2 Otorhinolaryngology Institute, Sun Yat-Sen University, Guangzhou 510080, Guangdong, P.R. China; 3 Department of Otolaryngology, The Sixth Affiliated Hospital of Sun Yat-Sen University, Guangzhou 510655, Guangdong, P.R. China

**Keywords:** ferritin light chain, ferritin heavy chain, tumor-associated macrophages, T regulatory cells, iron metabolism

## Abstract

Ferritin is the most important iron storage form and is known to influence tumor immunity. We previously showed that expression of ferritin light chain (FTL) and ferritin heavy chain (FTH1) subunits is increased in head and neck squamous cell carcinoma (HNSC). Here, we analyzed solid tumor datasets from The Cancer Genome Atlas and Genotype-Tissue Expression databases to investigate correlations between *FTL* and *FTH1* expressions and (i) patient survival, using univariate, multivariate, Kaplan-Meier and Receiver Operator Characteristic analysis; and (ii) tumor-infiltrating immune cell subsets, using the bioinformatics tools Estimation of Stomal and Immune cells in Malignant Tumor tissues, Microenvironment Cell Population-counter, Tumor Immune Estimation Resource, and Tumor Immunology Miner. We found that *FTL* and *FTH1* are upregulated and downregulated, respectively, in most of the human cancers analyzed. Tumor *FTL* levels were associated with prognosis in patients with lower grade glioma (LGG), whereas FTH1 levels were associated with prognosis in patients with liver hepatocellular carcinoma, HNSC, LGG, and kidney renal papillary cell carcinoma. In many cancers, *FTL* and *FTH1* levels was significantly positively correlated with tumor infiltration by tumor-associated macrophages and T regulatory cells. These results suggest an important role for *FTL* and *FTH1* in regulating tumor immunity to solid cancers.

## INTRODUCTION

Ferritin, the most important iron storage form in humans, is an essential nutrient that plays crucial roles in diverse cellular physiopathological processes, including regulation of the tumor microenvironment and immunometabolism [1, 2]. Ferritin is composed of two subunits: a light chain (FTL, 19 kDa) and a heavy chain (FTH, 21 kDa) [3]. We previously showed that FTH1 and FTL have positive linear correlation and the protein expression levels of both were higher in head and neck squamous cell carcinoma (HNSC) tumor tissues compared with normal tissues, and that elevated FTH1 was related to poorer prognosis [4].

Increasing evidence supports a role for iron metabolism in supporting immunity to various cancers [1, 5, 6]. Increases in intracellular iron initially promote T and B cell proliferation, but excessive iron levels ultimately induce oxidative stress-related cell death [7]. Reducing the iron content of the tumor microenvironment has been shown to improve the anti-tumor immune response in breast cancer [8], suggesting that iron chelation therapy in combination with conventional treatment modalities may be a promising strategy for cancer therapy [9]. However, the role played by ferritin in regulating the tumor microenvironment is unclear, and little is known about whether or how *FTL* and *FTH1* expression levels might correlate with tumor infiltration by diverse immune cell subsets. The Pan-Cancer Atlas, which is an essential resource for the development of precision medicine, seeks to reclassify cancers on the basis of their molecular similarities rather than their cellular origins. Along these lines, a comprehensive analysis of correlations between ferritin subunit levels and immune components in solid tumors could provide us with a better understanding of how iron metabolism influences tumor immunity.

In the present study, we analyzed the expressions of *FTL* and *FTH1* in various cancer RNA-sequencing datasets, and we performed a comprehensive analysis of correlations between *FTL* and *FTH1* levels and tumor-infiltrating immune cells, with the goal of uncovering a potential role for *FTL* and *FTH1* in tumor immunology. To this end, we analyzed datasets from The Cancer Genome Atlas (TCGA), a landmark cancer genomics database started in 2006 [10–12], and additionally employed a number of bioinformatics tools to assess the relationship between tumor *FTL* and *FTH1* levels and tumor infiltration by various immune cell subsets. These tools included Estimation of Stromal and Immune cells in Malignant Tumor tissues using Expression data (Estimate) [13], Microenvironment Cell Population (MCP)-counter [14], Tumor Immune Estimation Resource (TIMER), [15] and Tumor-Immune Miner (TIMINER) [16].

## RESULTS

### *FTL* and *FTH1* are upregulated and downregulated, respectively, in most human cancers assessed

**Figure 1:**
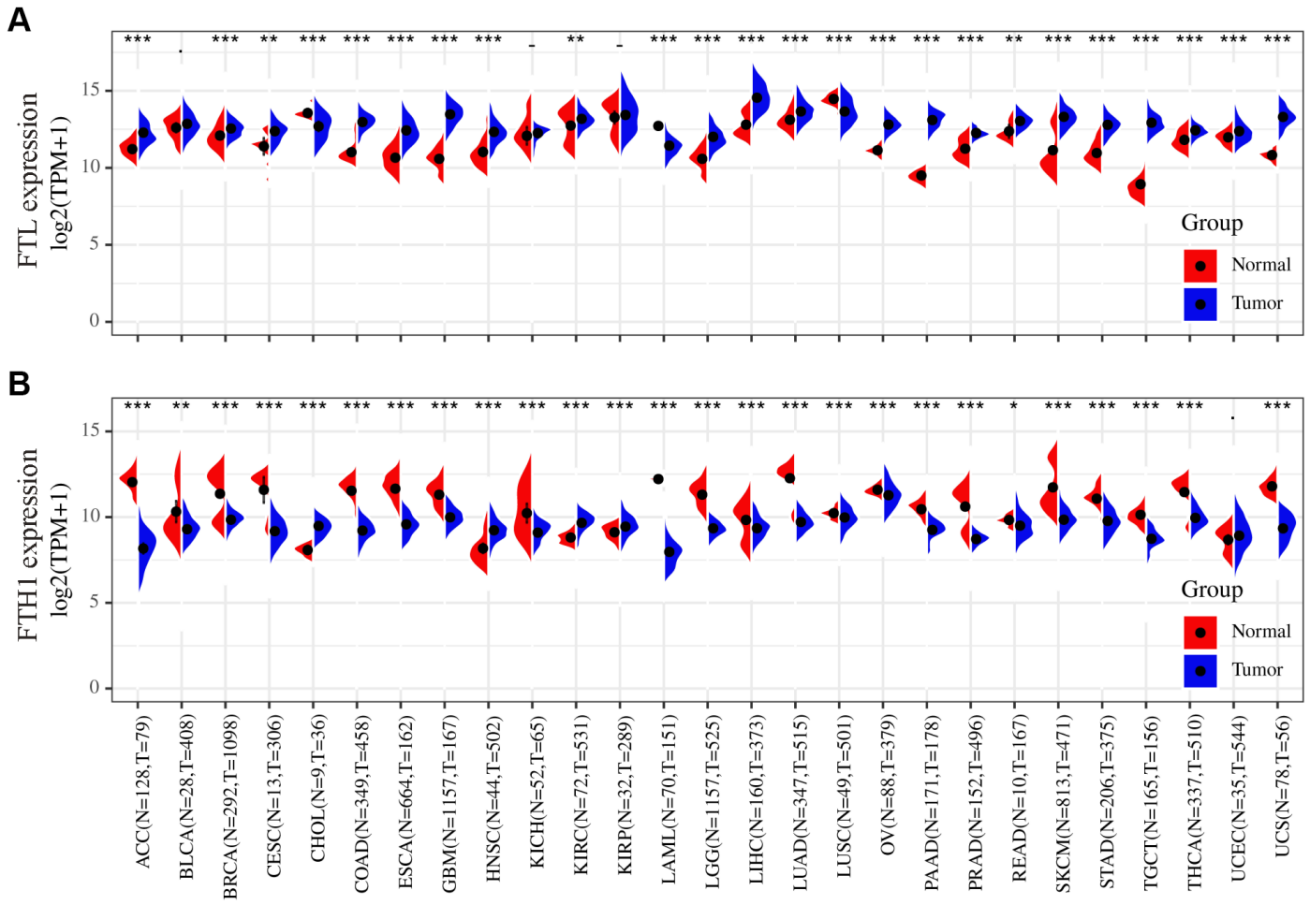
**Differences in the *FTL* and *FTH1* expressions between 27 tumors and corresponding normal tissues.** (**A**) *FTL* level was up-regulated in tumors of ACC, BRCA, CESC, COAD, ESCA, GBM, HNSC, KIRC, LGG, LIHC, LUAD, OV, PAAD, PRAD, READ, SKCM, STAD, TGCT, THCA, UCEC, UCS, down-regulated of CHOL, LAML, LUSC. (**B**) *FTH1* level was up-regulated in tumors of CHOL, HNSC, KIRC, KIRP, down-regulated of ACC, BRCA, BLCA, CESC, COAD, ESCA, GBM, KICH, LAML, LUSC, LGG, LIHC, LUAD, OV, PAAD, PRAD, READ, SKCM, STAD, TGCT, THCA, UCS. Datasets were from TCGA and GTEx. - not significant, * P < 0.05, ** P <0.01, *** P < 0.001.

To determine whether *FTL* and *FTH1* are differentially expressed in human cancers, we examined expression datasets from TCGA and GTEx databases. The results showed that *FTL* was upregulated in 21 of the 27 cancers analyzed (77.8%), downregulated in three cancers (11.1%; CHOL, LAML, LUSC), and not significantly changed in three cancers (11.1%; BLCA, KICH, KIRP) compared with normal tissues (Figure 1A). Conversely, *FTH1* was downregulated in 22 of the 27 cancers (81.5%), upregulated in four cancers (14.8%; CHOL, HNSC, KIRC, KIRP), and unchanged in only one cancer (3.7%; UCEC) (Figure 1B).

### *FTL* and *FTH1* levels are positively associated with poor prognosis in many human cancers

**Figure 2:**
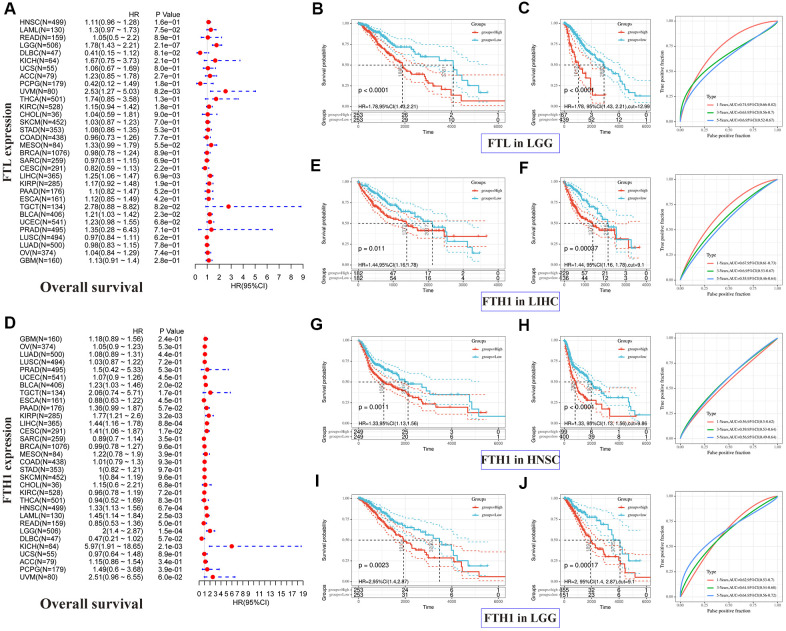
**Positive correlations between high *FTL* and *FTH1* expressions and poor OS in several tumors.** (**A**) Univariate analysis showed the positive association between high *FTL* level and poor OS in patients with LGG (P = 2.1 × 10^−7^), UVM (P = 8.2 × 10^−3^), LIHC (6.9 × 10^−3^), BLCA (P = 2.3 × 10^−2^). (**B**, **C**) High *FTL* mRNA level was related to poor OS in LGG (HR 1.78, 95% CI 1.43–2.21, P < 0.0001). (**D**) Univariate analysis showed the positive association between high *FTH1* level and poor OS in patients with BLCA (P = 0.2 × 10^−1^), KIRP (P = 3.2 × 10^−3^), LIHC (P = 8.8 × 10^−4^), CESC (P = 1.7 × 10^−2^), HNSC (P = 6.7 × 10^−4^), LAML (P = 2.5 × 10^−3^), LGG (P = 1.5 × 10^−4^), KICH (P = 2.1 × 10^−3^). (**E**–**J**) High *FTH1* mRNA level was related to poor OS in LIHC (HR 1.44, 95% CI 1.16–1.78, P = 1.1 × 10^−2^), HNSC (HR 1.33, 95% CI 1.13–1.56, P = 1.1 × 10^−3^), LGG (HR 2, 95% CI 1.4–2.87, P = 2.3 × 10^−3^). Only the tumors with P < 0.001 in univariate analysis will do the further Kaplan–Meier and ROC analysis.

Correlations between *FTL* and *FTH1* levels and patient prognosis were first examined separately by univariate survival analysis. *FTL* expression was significantly associated with OS in 4 cancer types (Figure 2A): LGG (hazard ratio [HR] 1.78, 95% confidence interval [CI] 1.43–2.21, P = 2.1 × 10^−7^), UVM (HR 2.53, 95% CI 1.27–5.03, P = 8.2 × 10^−3^), LIHC (HR 1.25, 95% CI 1.06–1.47, P = 6.9 × 10^−3^), and BLCA (HR 1.21, 95% CI 1.03–1.42, P = 2.3 × 10^−2^). *FTH1* expression was significantly associated with OS in eight cancer types (Figure 2D): BLCA (HR 1.23, 95% CI 1.03–1.46, P = 0.2 × 10^−1^), KIRP (HR 1.77, 95% CI 1.21–2.6, P = 3.2 × 10^−3^), LIHC (HR 1.44, 95% CI 1.16–1.78, P = 8.8 × 10^−4^), CESC (HR 1.41, 95% CI 1.06–1.87, P = 1.7 × 10^−2^), HNSC (HR 1.33, 95% CI 1.13–1.56, P = 6.7 × 10^−4^), LAML (HR 1.45, 95% CI 1.14–1.84, P = 2.5 × 10^−3^), LGG (HR 2, 95% CI 1.4–2.87, P = 1.5 × 10^−4^), and KICH (HR 5.97, 95% CI 1.91–18.65, P = 2.1 × 10^−3^). We then selected cancers in which *FTL* and *FTH1* levels were associated with OS at P < 0.001, and performed Kaplan–Meier analysis of OS in patients stratified into high and low expression groups by ROC curve analysis. The results suggested that high *FTL* (Figure 2B, 2C) or high *FTH1* (Figure 2E–2J) mRNA levels were each related to poor OS in several cancer types. Cox regression analysis further showed that FTL was an independent prognostic marker in LGG, UVM and LIHC (Supplementary Table 2).

**Figure 3:**
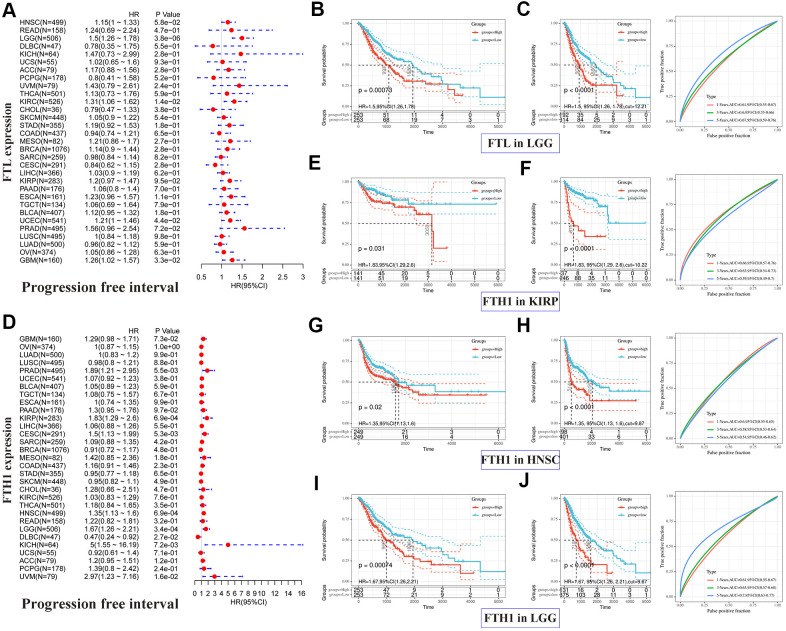
**Positive correlations between high *FTL* and *FTH1* expressions and poor PFI in several tumors.** (**A**) Univariate analysis showed the positive association between high *FTL* level and poor PFI in patients with LGG (P = 3.8 × 10^−6^), KIRC (P = 1.4 × 10^−2^), UCEC (4.4 × 10^−2^), GBM (P = 3.3 × 10^−2^). (**B**, **C**) High *FTL* mRNA level was related to poor OS in LGG (HR 1.5, 95% CI 1.26–1.78, P = 7.3 × 10^−4^). (**D**) Univariate analysis showed the positive association between high *FTH1* level and poor PFI in patients with PRAD (P = 5.5 × 10^−3^), KIRP (P = 6.9 × 10^−4^), CESC (P = 5.3 × 10^−3^), HNSC (P = 6.9 × 10^−4^), LGG (P = 3.4 × 10^−4^), KICH (P = 7.2 × 10^−3^), UVM (P = 1.6 × 10^−2^), and the negative association with DLBC (P = 2.7 × 10^−2^). (**E**–**J**) High *FTH1* mRNA level was related to poor PFI in KIRP (HR 1.83, 95% CI 1.29–2.6, P = 3.1 × 10^−2^), HNSC (HR 1.35, 95% CI 1.13–1.6, P = 2 × 10^−2^), LGG (HR 1.67, 95% CI 1.26–2.21, P = 7.4 × 10^−4^). Only the tumors with P < 0.001 in univariate analysis will do the further Kaplan–Meier and ROC analysis.

Next, we performed the same analyses to examine correlations between *FTL* and *FTH1* expressions and PFI and, not surprisingly, obtained results similar to the OS analysis. *FTL* expression was significantly associated with PFI in four cancer types (Figure 3A): LGG (HR 1.5, 95% CI 1.26–1.78, P = 3.8 × 10^−6^), KIRC (HR 1.31, 95% CI 1.06–1.62, P = 1.4 × 10^−2^), UCEC (HR 1.21, 95% CI 1.00–1.46, P = 4.4 × 10^−2^), and GBM (HR 1.26, 95% CI 1.02–1.57, P = 3.3 × 10^−2^). *FTH1* expression was significantly associated with PFI in eight cancer types (Figure 3D): PRAD (HR 1.89, 95% CI 1.21–2.95, P = 5.5 × 10^−3^), KIRP (HR 1.83, 95% CI 1.29–2.6, P = 6.9 × 10^−4^), CESC (HR 1.5, 95% CI 1.13–1.99, P = 5.3 × 10^−3^), HNSC (HR 1.35, 95% CI 1.13–1.6, P = 6.9 × 10^−4^), LGG (HR 1.67, 95% CI 1.26–2.21, P = 3.4 × 10^−4^), DLBC (HR 0.47, 95% CI 0.24–0.92, P = 2.7 × 10^−2^), KICH (HR 5, 95% CI 1.55–16.19, P = 7.2 × 10^−3^), and UVM (HR 2.97, 95% CI 1.23–7.16, P = 1.6 × 10^−2^). As was observed for OS, poor PFI was significantly associated with high *FTL* (Figure 3B, 3C) or high *FTH1* (Figure 3E–3J) mRNA levels in some cancers.

### *FTL* and *FTH1* levels correlate positively with immune checkpoint markers

**Figure 4:**
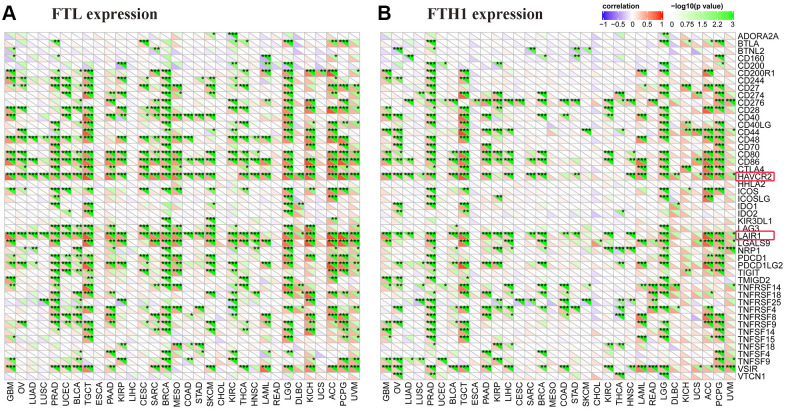
***FTL* and *FTH1* levels were positively correlated with some of 47 distinct immune-related markers in 32 solid tumors.** (**A**) *FTL* level was positively related to some immune-related markers, of which the most 2 were *TIM-3* (26/32, 81.3%) and *LAIR1* (26/32, 81.3%). Besides, *FTL* level was positively related to *PD-1* in 14/32 cancers (43.8%), *PD-L1* in 10/32 (31.3%), *CTLA4* in 13/32 (40.6%), and *LAG3* in 13/32 (40.6%). (**B**) *FTH1* level was positively related to some immune-related markers, of which the most 2 were *TIM-3* (20/32, 62.5%) and *LAIR1* (19/32, 59.4%). Besides, *FTH1* level was positively related to *PD-1* in 4/32 (12.5%) cancers, *PD-L1* in 9/32 (28.1%), *CTLA4* in 7/32 (21.9%), and *LAG3* in 2/32 (6.3%).

To clarify the potential role of *FTL* and *FTH1* in tumor immunity, we next examined correlations between the expressions of *FTL* and *FTH1* and 47 distinct immune-related markers using Pearson Correlation Coefficient analysis. As shown in Figure 4A, we found that *FTL* levels were positively related to *PD-1* in 14/32 cancers (43.8%), *PD-L1* in 10/32 (31.3%), *CTLA4* in 13/32 (40.6%), *TIM-3* in 26/32 (81.3%), *LAG3* in 13/32 (40.6%), and *LAIR1* in 26/32 (81.3%). *FTH1* levels (Figure 4B) were positively related to *PD-1* in 4/32 (12.5%) cancers, *PD-L1* in 9/32 (28.1%), *CTLA4* in 7/32 (21.9%), *TIM-3* in 20/32 (62.5%), *LAG3* in 2/32 (6.3%), and *LAIR1* in 19/32 (59.4%). Interestingly, *TIM-3* was positively related to *FTL* and *FTH1* in the most cancers, so does *LAIR1* (Figure 4A, 4B).

### *FTL* and *FTH1* levels correlate positively with immune score in most cancers

**Figure 5:**
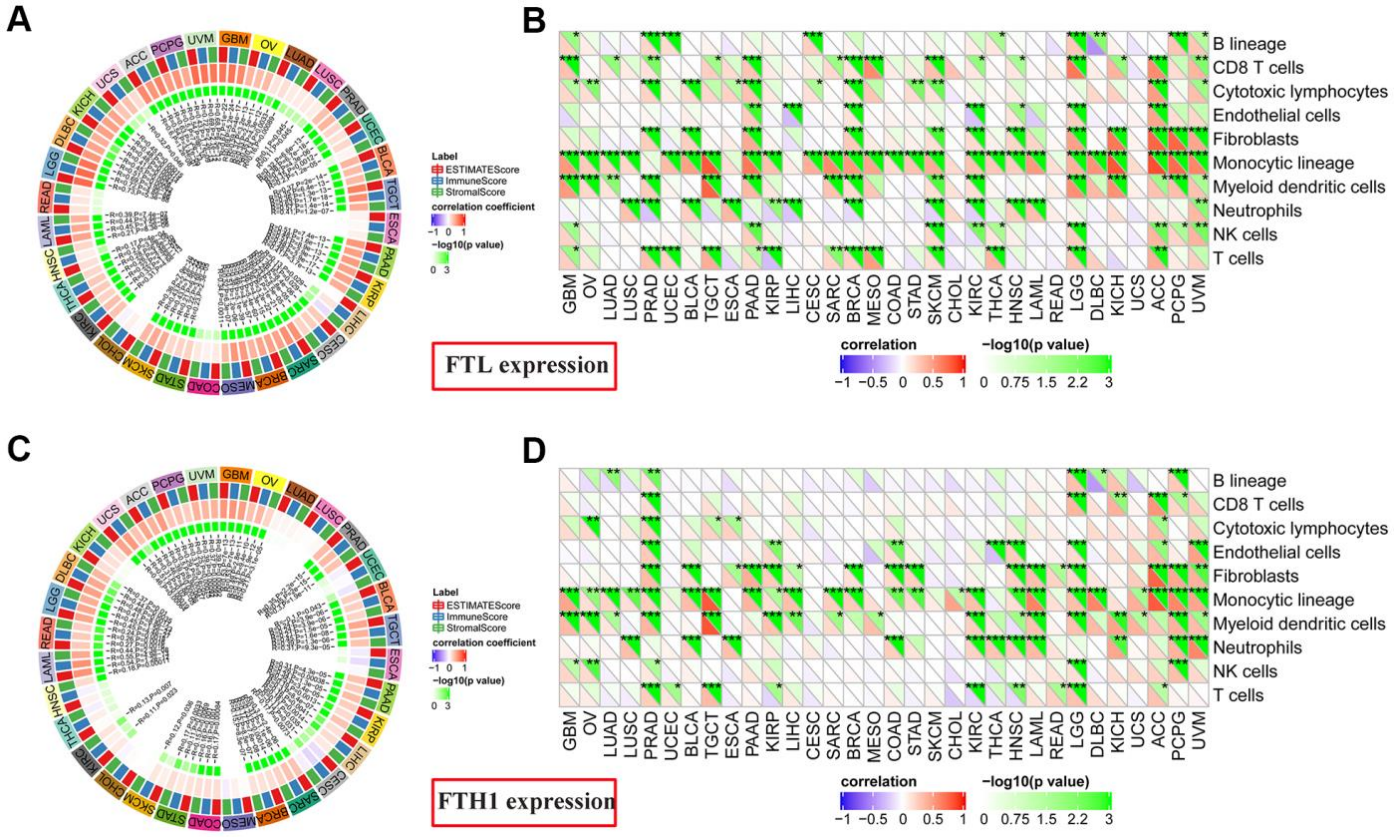
**Correlations between *FTL* and *FTH1* expression levels and immune cell infiltration determined using Estimate Immune Score and MCP-counter analyses.** (**A**) *FTL* level was correlated positively with Immune Score in most cancers (28/32, 87.5%), with only READ, ESCA, COAD, and CHOL displaying no correlation. (**B**) MCP-counter analysis revealed positive associations between *FTL* expression and several cell subsets, of which the most 3 were monocytic lineage cells (26/32, 81.3%), CD8 T cell (15/32, 46.9%), and myeloid dendritic cells in (15/32, 46.9%). (**C**) *FTH1* level was positively correlated with Immune Score in most cancers (22/32, 68.8%), negatively correlated in 1 (3.1%; CESC), and was not correlated in 9/32 (28.1%; KICH, LUAD, LUSC, UCEC, ESCA, MESO, CHOL, THCA, HNSC). (**D**) MCP-counter analysis revealed positive associations between *FTH1* expression and several cell subsets, of which the most 3 were monocytic lineage cells (24/32, 75.0%), myeloid dendritic cells (17/32, 53.1%), and fibroblasts (16/32, 50.0%).

Given these findings that expressions of both *FTL* and *FTH1* correlated strongly with the expression of immune-related markers in many cancers (Figure 4A, 4B), we next investigated correlations with the Immune Score, which itself is known to correlate strongly with patient survival [14]. *FTL* expression (Figure 5A) correlated positively with Immune Score in most cancers (28/32, 87.5%), with only READ, ESCA, COAD, and CHOL displaying no correlation. Similarly, *FTH1* expression (Figure 5C) was positively correlated with Immune Score in most cancers (22/32, 68.8%), negatively correlated in 1 (3.1%; CESC), and was not correlated in 9/32 (28.1%; KICH, LUAD, LUSC, UCEC, ESCA, MESO, CHOL, THCA, HNSC).

### *FTL* and *FTH1* levels correlate positively with infiltration of Tregs and TAMs in most cancers

To determine the source(s) of the correlation between *FTL* and *FTH1* levels and Immune Score in most cancers, we examined correlations between expression and tumor-infiltrating cell subsets using MCP-counter, TIMER, and TIMINER. First, MCP-counter analysis revealed positive associations between *FTL* expression (Figure 5B) and several cell subsets, predominantly monocytic lineage cells (26/32, 81.3%), CD8 T cell (15/32, 46.9%), and myeloid dendritic cells in (15/32, 46.9%), while *FTH1* expression (Figure 5D) was most clearly associated with monocytic lineage cells (24/32, 75.0%), myeloid dendritic cells (17/32, 53.1%), and fibroblasts (16/32, 50.0%).

**Figure 6:**
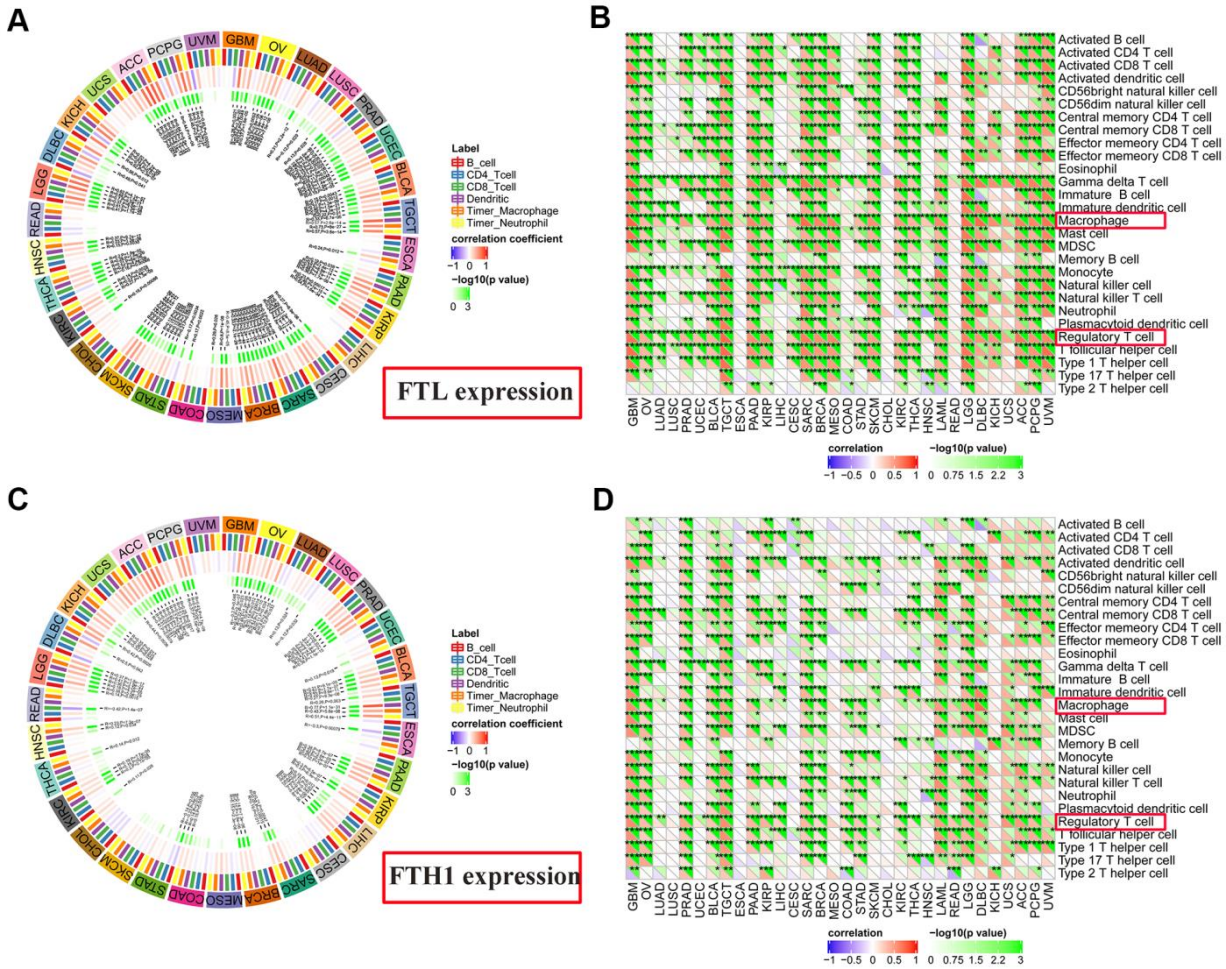
**Correlations between *FTL* and *FTH1* expressions and immune cell infiltration determined using TIMER and TIMINER analyses.** (**A**) TIMER analysis of the association between *FTL* expression and B cells, CD4^+^ T cells, CD8^+^ T cells, dendritic cells, TAMs and neutrophils in 31 solid tumors. (**B**) TIMINER analysis of the positive association between *FTL* expression and 28 immune cell subtypes, of which the most 3 were gamma delta T cells (29/32, 90.6%), Tregs (28/32, 87.5%), and TAMs (28/32, 87.5%). (**C**) TIMER analysis of the association between *FTH1* expression and B cells, CD4^+^ T cells, CD8^+^ T cells, dendritic cells, TAMs and neutrophils in 31 solid tumors. (**D**) TIMINER analysis of the positive association between *FTH1* expression and 28 immune cell subtypes, of which the most 3 were Tregs (25/32, 78.1%), TAMs (24/32, 75.0%), and activated dendritic cells (24/32, 75.0%).

TIMER analysis revealed that *FTL* expression and CD4^+^ T cells (Figure 6A) were positively correlated in 11/31 (35.5%) cancers, negatively correlated in 2/31 (6.5%), and was not correlated in 18/31 (58.1%). *FTL* expression and CD8^+^ T cells (Figure 6A) were positively correlated in 15/31 (48.4%), negatively correlated in 3/31 (9.7%), and not correlated in 13/31 (41.9%). Similarly, *FTH1* and CD4^+^ T cells (Figure 6C) were positively correlated in 7/31 (22.6%), negatively correlated in 2/31 (6.5%), and not correlated in 22/31 (71.0%). *FTH1* and CD8^+^ T cells (Figure 6C) were positively correlated in 7/31 (22.6%), negatively correlated in 3/31 (9.7%), and not correlated in 21/31 (67.7%).

Finally, TIMINER was employed to further analyze the associations between *FTL* and *FTH1* expressions and other subtypes of tumor-infiltrating immune cells. Interestingly, although the use of both MCP-counter and TIMER identified positive correlations between *FTL* and *FTH1* expressions and CD4^+^ or CD8^+^ T cells in fewer than 50% of the cancer types examined, analysis of TIMINER identified a much higher rate of association between *FTL* and *FTH1* expressions and tumor-infiltrating immune cell subsets. Thus, *FTL* expression (Figure 6B) was associated most strongly with gamma delta T cells in 29/32 (90.6%) cancers, Tregs in 28/32 (87.5%), and TAMs in 28/32 (87.5%), while *FTH1* expression (Figure 6D) was most strongly associated with Tregs in 25/32 (78.1%), TAMs in 24/32 (75.0%), and activated dendritic cells in 24/32 (75.0%). Furthermore, there is a positive correlation between FTH1 and FTL levels and CD163, the scavenger marker of M2 TAMs (Supplementary Figure 1). These data demonstrated that both Tregs and TAMs were positively correlated with expression of ferritin subunits in most cancers.

## DISCUSSION

Increasing recognition that iron metabolism plays complex roles in cancer biology has fueled intense interest in the iron storage protein ferritin [1, 17], particularly for its roles as a mediator of the Fenton reaction in mitochondrial oxidative phosphorylation, as an integral player in the iron-dependent cell death pathway ferroptosis, and its potential utility as a drug nanocarrier [18]. FTH1 specifically binds to transferrin receptor 1, which is upregulated in most malignant cells [19, 20], thereby allowing ferritin-mediated targeted drug delivery [21] with the goal of enhancing efficacy [20, 22, 23]. Iron is an essential element in supporting cell proliferation and often accumulates in cancer cells; however, excessive levels of Fe^2+^ can promote the Fenton reaction, production of reactive oxygen species, and cell death through apoptosis [17] and ferroptosis [18].

Upregulation of ferritin has been shown to correlate with poor prognosis in several cancers, including HNSC, non-small cell lung cancer, and liver cancer [24, 25]. Nevertheless, apart from two studies showing that FTL is elevated in GBM and colorectal cancer [26, 27], little is known about the expressions of *FTL* and *FTH1* in cancer. In the present study, we found that *FTL* is upregulated in most of the 27 cancers examined, which is consistent with findings in other cancers and is likely related to the essential role of ferritin in cell proliferation [1, 28, 29]. We also showed that *FTL* expression is positively related to poor prognosis in seven cancers, particularly LGG, and that *FTL* expression is elevated significantly in six of those cancers. FTL can accelerate the storage efficiency of Fe^3+^ [28, 29]. Correlations between FTL protein level and poor prognosis have been identified in node-negative breast cancer [30] and colorectal cancer [26], but these findings were not confirmed in our study. This discrepancy may be due to the different patient selection criteria. For example, the study by Gabriel et al. analyzed only patients with node-negative breast cancer [30], whereas the lymph node metastasis status was not a factor in the breast cancer dataset evaluated here.

In contrast to *FTL*, *FTH1* expression was downregulated in 22 and upregulated in four of the 27 cancers examined in our study. We found that high *FTH1* expression is positively related to poor prognosis in 11 cancers, including HNSC, which is consistent with our previous study [4]. Among these 11 cancers, the *FTH1* level was significantly downregulated in nine cancers and significantly upregulated in two (KIRP, HNSC) compared with normal tissues. *FTH1* possesses ferroxidase activity and converts Fe^2+^ into Fe^3+^, its combination with FTL can efficiently reduce the toxicity of Fe^2+^ [28, 29]. In cancer cells, *FTH1* is thought to be a bifunctional molecule. Flavia et al. [31] found that increased intracellular levels of *FTH1* can enhance P53 expression and reduce the proliferation of non-small cell lung cancer. However, Salatino et al. [29] showed that *FTH1* is critical for proper functioning of the antioxidant system in ovarian cancer cells, suggesting that inhibition of *FTH1* may improve cisplatin-induced cytotoxicity. In addition, the subcellular localization of *FTH1* can affect its functions. Liu showed that a high cytoplasmic *FTH1* level correlates with a favorable prognosis, whereas nuclear *FTH1* is an adverse indicator, in triple negative breast cancer patients [32]. It is still unclear which factors control the translocation of *FTH1*. Clearly, *FTH1* has multiple complex functions in cancer cells, and a broader understanding of its roles is urgently needed.

Advances in our understanding of anti-tumor immunity have made targeted immunotherapies the most promising treatment methods for many cancers [33, 34]. In the present study, we found that expressions of *FTL* and *FTH1* correlated positively with levels of the immune checkpoint proteins *TIM-3* and *LAIR1*, both of which can suppress tumor immunity [35–38]. *TIM-3* is a negative regulator of cytotoxic and helper T cells and can promote the formation of a suppressive tumor microenvironment through the *TIM-3*–*galectin-9* pathway [36, 39]. *LAIR1* is widely expressed in immune cells where it negatively regulates cell activation through interaction with several ligands, especially collagen [37]. However, to our knowledge, the present study is the first to reveal these positive correlations between *TIM-3* and *LAIR1* levels and *FTL* and *FTH1* levels. Moreover, few studies have assessed the influence of *TIM-3* and *LAIR1* on iron metabolism. We speculate that *FTL* and *FTH1* may influence *TIM-3* and *LAIR* levels by affecting immune cell infiltration into the tumor.

In addition to T cells, we investigated potential correlations between *FTL* and *FTH1* expressions in solid tumors and infiltration of several other pivotal immune cells. Intriguingly, *FTL* and *FTH1* levels are positively correlated with most of immune cells in some tumors, especially in LGG. However, both of them are positively correlated with the poor prognosis of LGG. Iron is essential for cell metabolism and proliferation [17], however, each immune cell type has its own metabolic adaptation in the tumor microenvironment [40]. We consider that the influence extent of *FTL* and *FTH1* on different immune cells should be various. In other words, the increasing level of immunosuppressive cells, like M2 and Treg, could completely offset the effect of anti-tumor immune cells. However, this needs to be confirmed in a large number of future studies.

Moreover, our analyses revealed positive associations between *FTL* and *FTH1* expressions and infiltration of both cell types in most cancers. Mounting evidence suggests that TAMs exhibit high plasticity and can switch between the anti-tumoral M1 subtype and the pro-tumoral M2 subtype, the latter of which is most abundant in solid tumors [41, 42]. Ricolleau reported that *FTL* is mainly stored in M2-like TAMs in node-negative breast cancer [43]. Cronin showed that M1-like macrophages can sequester iron and produce reactive oxygen species, and that M2-like macrophages release iron to promote tumor progression [2]. On the basis of these studies, we speculate that the positive correlation between *FTL* and *FTH1* levels and TAMs may be due to the high demand for ferritin in M2 TAMs. However, whether *FTL* or *FTH1* inhibition can induce M2-to-M1 repolarization will need to be investigated in future studies.

Finally, the present study shows that both *FTL* and *FTH1* levels are positively correlated with Tregs infiltration in most cancers [44]. A similar correlation has recently been reported in melanoma [44]. Gray showed that melanoma cells secreting *FTH1* could activate Tregs to produce interleukin-10 and suppress the immune response *in vitro* [45]. As noted, the present study is the first to propose a positive correlation between *FTL* and *FTH1* expressions and Treg infiltration in most solid tumors through analysis of mRNA and clinicopathological data from TCGA. While a robust source of iron is necessary for metabolic and redox reactions to support the proliferation and effector functions of T cells [46], excessive levels of intracellular iron could induce cell death via induction of oxidative stress [7]. The present study suggests a need for comparable iron and ferritin homeostasis in Tregs, which are the most important immunosuppressive T-cell subset. To the best of our knowledge, no studies have yet examined whether targeted *FTL* or *FTH1* therapy could influence Treg infiltration by decreasing ferritin expression, which holds promise as a new mechanism of iron-based therapy. Whether such therapy could also enhance the activity of immunotherapies is another question that should be addressed in future studies.

In summary, we have shown that the expressions of *FTL* and *FTH1* mRNA is increased and decreased, respectively, in most of the 27 solid cancer types examined. Furthermore, both ferritin subunits may have important physiological functions in TAMs and Tregs, and thus may play key roles in tumor immunity. Our results suggest that therapies targeting iron and ferritin homeostasis could be developed as potential treatments to synergize with specific immunotherapies to enhance anti-tumor responses.

## MATERIALS AND METHODS

### TCGA and GTEx datasets

The expression levels of genes in various types of cancers were downloaded from TCGA (https://www.cancer.gov/about-nci/organization/ccg/research/structural-genomics/tcga/, OV: ovarian serous cystadenocarcinoma, LUAD: lung adenocarcinoma, LUSC: lung squamous cell carcinoma, PRAD: prostate adenocarcinoma; UCEC: uterine corpus endometrial carcinoma, BLCA: bladder urothelial carcinoma, TGCT: testicular germ cell tumor, ESCA: esophageal carcinoma, PAAD: pancreatic adenocarcinoma, KIRP: kidney renal papillary cell carcinoma, LIHC: liver hepatocellular carcinoma, CESC: cervical squamous cell carcinoma and endocervical adenocarcinoma, SARC: sarcoma, BRCA: breast invasive carcinoma; MESO: mesothelioma, COAD: colon adenocarcinoma, STAD: stomach adenocarcinoma, SKCM: skin cutaneous melanoma, CHOL: cholangiocarcinoma, KIRC: kidney renal clear cell carcinoma, THCA: thyroid carcinoma, THYM: thymoma, HNSC, READ: rectum adenocarcinoma, LGG, DLBC: lymphoid neoplasm diffuse large B-cell lymphoma, KICH: kidney chromophobe, UCS: uterine carcinosarcoma, ACC: adrenocortical carcinoma, PCPG: pheochromocytoma and paraganglioma, UVM: uveal melanoma, GBM: glioblastoma multiforme). Gene expression datasets for the corresponding normal tissues were downloaded from the Genotype-Tissue Expression (GTEx) program (https://www.gtexportal.org/home/). Patients’ clinical information were organized in Supplementary Table 1. All gene expression data were normalized and are presented here as log2 of the transcript control per million +1.

### Prognosis analysis

Overall survival (OS) and progression-free interval (PFI) data were obtained from TCGA. Univariate survival analysis was used to analyze correlations between *FTL* and *FTH1* levels and OS and PFI separately, and cancers with significant associations at p<0.001 were further analyzed using the Kaplan–Meier method. Patients were assigned to low and high *FTL* and *FTH1* expression groups using receiver operating characteristic (ROC) curve analysis, with the median expression level as the cutoff value. A multivariate Cox proportional hazard model was performed to evaluate the independent prognostic variables.

### Immune-related markers

Expression datasets for 47 immune-related markers were also obtained from TCGA. The markers included pivotal immune checkpoint proteins such as programmed death-1 (*PD-1*/*PDCD1*), programmed cell death-1 ligand 1 (*PD-L1*/*CD274*), cytotoxic T lymphocyte-associated protein 4 (*CTLA4*), T-cell immunoglobulin domain and mucin domain-3 (*TIM-3*/*HAVCR2*), lymphocyte-activation gene 3 (*LAG3*), and leukocyte-associated immunoglobulin-like receptor 1 (*LAIR1*).

### Estimate Score and MCP-counter

Expression data to identify cell subsets were downloaded from TCGA. The Immune Score, Stromal Score, and Estimate Score were calculated using R (version 3.6.3), RStudio (version 1.2.5033), and R Estimate package (version 1.0.13). The MCP-counter data were analyzed using R MCP-counter package (version 1.2.0).

### TIMER and TIMINER

TIMER (https://cistrome.shinyapps.io/timer/) is a deconvolution tool to calculate the abundance of tumor-infiltrating immune cell subsets, including B cells, CD4^+^ T cells, CD8^+^ T cells, neutrophils, macrophages, and dendritic cells, in various human cancers. TIMINER (https://icbi.i-med.ac.at/software/timiner/timiner.shtml) is a computational framework that is based on the pre-ranked Gene Set Enrichment Analysis approach and was used here to define gene sets from 28 immune cell subtypes.

### Statistical analysis

Differences in FTL and FTH1 expressions between tumor and normal tissues were compared using Student’s t-test. Survival data were analyzed by univariate and Kaplan–Meier analysis of patient populations stratified by ROC curve analysis. Associations between *FTL* and *FTH1* expressions and tumor-infiltrating cells or immune-related markers were analyzed using Pearson’s Correlation Coefficient. P < 0.05 was considered statistically significant.

## Supplementary Material

Supplementary Figure 1

Supplementary Tables
