# Supplementary material for: Comprehensive analysis of ferritin subunits expression and positive correlations with tumor-associated macrophages and T regulatory cells infiltration in most solid tumors
Source: Aging (Albany NY). 2021 Apr 16;13(8):11491–506. doi: 10.18632/aging.202841 (PMC8109065; doi:10.18632/aging.202841)
Supplement: Supplementary Figure 1 [file aging-13-202841-s001.pdf]

SUPPLEMENTARY FIGURE

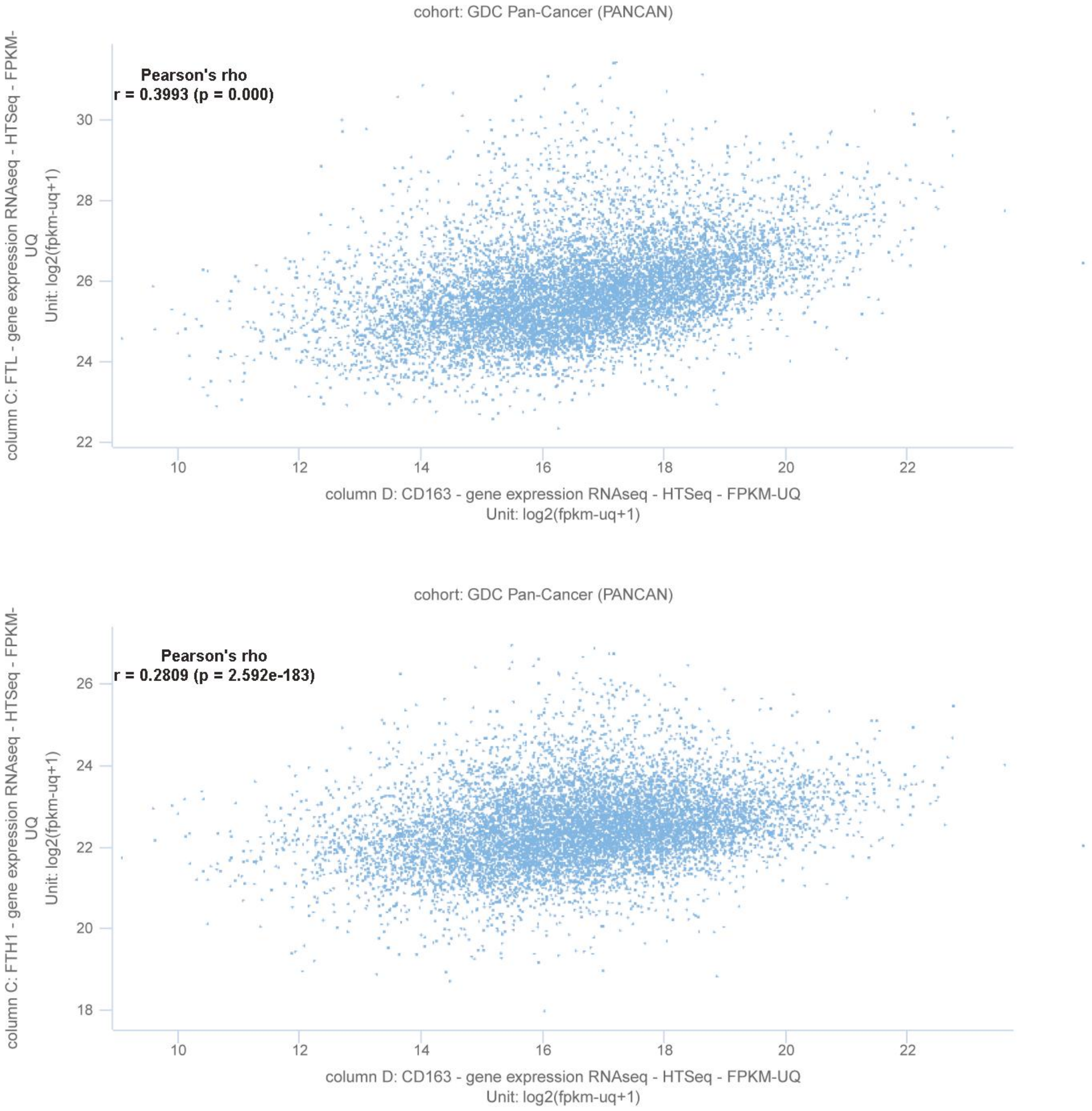

Supplementary Figure 1. The positive correlations between FTH1 and FTL levels and CD163.
