# Supplementary material for: Comprehensive analysis of ferritin subunits expression and positive correlations with tumor-associated macrophages and T regulatory cells infiltration in most solid tumors
Source: Aging (Albany NY). 2021 Apr 16;13(8):11491–506. doi: 10.18632/aging.202841 (PMC8109065; doi:10.18632/aging.202841)
Supplement: Supplementary Tables [file aging-13-202841-s002.pdf]

SUPPLEMENTARY TABLES

Supplementary Table 1. Baseline demographic and clinical characteristics of the patients in this study.

	n (%)
**Tumor**	10147(100)
Gender	
Male/ Female	4810 (47.4)/ 5337 (52.6)
Age (years at diagnosis)	
Mean	59.1
Range	10~90
**Samples for COX analysis**	3068(100)
Gender	
Male/ Female	1814 (59.1)/ 1254 (40.9)
Age (years at diagnosis)	
Mean	57.1
Range	14~90
T-stages	
T0/ T1	3(0.1)/ 679(22.1)
T2/ T3	515(16.8)/ 543(17.7)
T4/ Not reported	298(9.7)/ 1030(33.6)
N-stages	
N0/ N1	983(32.0)/ 221(7.2)
N2/ N3	255(8.3)/ 16(0.5)
Not reported	1593(51.9)
M-stages	
M0/ M1	998(32.5)/ 46(1.5)
Not reported	2024(66.0)
Clinical-stages	
I/ II	637(20.8)/ 483(15.7)
III/ IV	519(16.9)/ 482(15.7)
Not reported	947(30.9)
**Normal**	6678(100)
Gender	
Male/ Female	4074 (61.0)/ 2604 (39.0)
Age (years at diagnosis)	
Mean	61.4
Range	15~90

n. number of patients.

Supplementary Table 2. Correlation of FTH1 and FTL and common clinicopathological factors with patients' overall survival in the TCGA datasets.

		LGG	UVM	LIHC	BLCA	KIRP	CESC	HNSC	LAML	KICH
Likelihood ratio test	p	1E-15	0.002	0.00002	0.007	8e-07	0.04	4e-06	8e-05	8e-04
Wald test	p	4E-16	0.02	0.00003	0.008	0.002	0.01	5e-06	2e-04	1
Score (logrank) test	p	2E-16	0.005	0.000005	0.004	3e-13	0.009	2e-08	1e-04	4E-07
FTH1	p	0.692549	0.79707	0.2346	0.6144	0.95248	0.85855	0.10267	0.101408	0.999
	HR	0.9269	1.166	1.186	1.0885	0.9733	1.0400	1.4491	1.4170	8.537e-05
FTL	p	0.000132	0.01089	0.0344	0.5750	0.27542	0.77144	0.50358	0.486831	0.998
	HR	1.6298	6.087	1.294	0.9122	1.4923	0.9316	0.8860	0.8617	2.775e+11
Age	p	1.21E-13	0.00105	0.3568	0.1250	0.02366	0.41176	0.36534	0.000221	0.996
	HR	1.0001	1	1.000	1.0001	0.9999	1.0000	1.0000	1.0001	1.006e+00
Gender	p	0.602136	0.47593	0.5297	0.0782	0.87147	NA	0.06813	0.513440	0.994
	HR	0.9082	0.63	1.178	1.6040	0.8682	NA	1.7187	1.1610	1.314e-44
M-stages	p	NA	NA	0.6672	0.5218	0.00324	NA	0.00577	NA	0.999
	HR	NA	NA	0.742	1.4169	56.9888	NA	21.0568	NA	1.138e-06
N-stages	p	NA	NA	0.5869	0.3838	0.68099	0.00384	9.88e-06	NA	0.993
	HR	NA	NA	1.729	1.2352	0.7407	2.8817	2.0615	NA	1.294e+38
T-stages	p	NA	0.78393	0.3872	0.1743	0.12485	0.04467	0.00171	NA	0.999
	HR	NA	1.235	1.448	1.3953	0.2999	1.9518	1.7799	NA	9.227e+10
Clinical stages	p	NA	0.46687	0.5739	0.5797	0.11381	0.10909	0.00527	NA	0.999
	HR	NA	2.024	1.313	1.2045	3.2560	0.5947	0.5374	NA	2.570e-05

HR: hazard ration.
